# Language, Motor Ability and Related Deficits in Children at Familial Risk of Schizophrenia or Bipolar Disorder

**DOI:** 10.1093/schbul/sbae181

**Published:** 2024-10-28

**Authors:** Marta Schiavon, Birgitte K Burton, Nicoline Hemager, Aja N Greve, Katrine S Spang, Ditte Ellersgaard, Kerstin Jessica Plessen, Jens Richardt M Jepsen, Anne A E Thorup, Thomas Werge, Merete Nordentoft, Ron Nudel

**Affiliations:** CORE–Copenhagen Research Center for Mental Health, Mental Health Center Copenhagen, Copenhagen University Hospital, 2900 Copenhagen, Denmark; CORE–Copenhagen Research Center for Mental Health, Mental Health Center Copenhagen, Copenhagen University Hospital, 2900 Copenhagen, Denmark; iPSYCH, The Lundbeck Foundation Initiative for Integrative Psychiatric Research, 8210 Aarhus, Denmark; CORE–Copenhagen Research Center for Mental Health, Mental Health Center Copenhagen, Copenhagen University Hospital, 2900 Copenhagen, Denmark; iPSYCH, The Lundbeck Foundation Initiative for Integrative Psychiatric Research, 8210 Aarhus, Denmark; Mental Health Center for Child and Adolescent Psychiatry–Research unit, Mental Health Services in the Capital Region of Denmark, 2900 Copenhagen, Denmark; Department of Psychology, University of Copenhagen, 1357 Copenhagen, Denmark; iPSYCH, The Lundbeck Foundation Initiative for Integrative Psychiatric Research, 8210 Aarhus, Denmark; Psychosis Research Unit, Department of Clinical Medicine, Aarhus University Hospital–Psychiatry, 8200 Aarhus, Denmark; Department of Clinical Medicine, Faculty of Health, 8200 Aarhus University, Aarhus, Denmark; iPSYCH, The Lundbeck Foundation Initiative for Integrative Psychiatric Research, 8210 Aarhus, Denmark; Mental Health Center for Child and Adolescent Psychiatry–Research unit, Mental Health Services in the Capital Region of Denmark, 2900 Copenhagen, Denmark; CORE–Copenhagen Research Center for Mental Health, Mental Health Center Copenhagen, Copenhagen University Hospital, 2900 Copenhagen, Denmark; iPSYCH, The Lundbeck Foundation Initiative for Integrative Psychiatric Research, 8210 Aarhus, Denmark; iPSYCH, The Lundbeck Foundation Initiative for Integrative Psychiatric Research, 8210 Aarhus, Denmark; Mental Health Center for Child and Adolescent Psychiatry–Research unit, Mental Health Services in the Capital Region of Denmark, 2900 Copenhagen, Denmark; Department of Psychiatry, Division of Child and Adolescent Psychiatry, Hospital University Lausanne and Lausanne University, 1004 Lausanne, Switzerland; CORE–Copenhagen Research Center for Mental Health, Mental Health Center Copenhagen, Copenhagen University Hospital, 2900 Copenhagen, Denmark; iPSYCH, The Lundbeck Foundation Initiative for Integrative Psychiatric Research, 8210 Aarhus, Denmark; Mental Health Center for Child and Adolescent Psychiatry–Research unit, Mental Health Services in the Capital Region of Denmark, 2900 Copenhagen, Denmark; Center for Neuropsychiatric Schizophrenia Research and Center for Clinical Intervention and Neuropsychiatric Schizophrenia Research, Mental Health Services in the Capital Region of Denmark, 2600 Glostrup, Denmark; iPSYCH, The Lundbeck Foundation Initiative for Integrative Psychiatric Research, 8210 Aarhus, Denmark; Mental Health Center for Child and Adolescent Psychiatry–Research unit, Mental Health Services in the Capital Region of Denmark, 2900 Copenhagen, Denmark; Department of Clinical Medicine, Faculty of Health and Medical Sciences, University of Copenhagen, 2200 Copenhagen, Denmark; iPSYCH, The Lundbeck Foundation Initiative for Integrative Psychiatric Research, 8210 Aarhus, Denmark; Department of Clinical Medicine, Faculty of Health and Medical Sciences, University of Copenhagen, 2200 Copenhagen, Denmark; Institute of Biological Psychiatry, Mental Health Centre Sct. Hans, Mental Health Services Copenhagen, 4000 Roskilde, Denmark; CORE–Copenhagen Research Center for Mental Health, Mental Health Center Copenhagen, Copenhagen University Hospital, 2900 Copenhagen, Denmark; iPSYCH, The Lundbeck Foundation Initiative for Integrative Psychiatric Research, 8210 Aarhus, Denmark; Department of Psychology, University of Copenhagen, 1357 Copenhagen, Denmark; CORE–Copenhagen Research Center for Mental Health, Mental Health Center Copenhagen, Copenhagen University Hospital, 2900 Copenhagen, Denmark; iPSYCH, The Lundbeck Foundation Initiative for Integrative Psychiatric Research, 8210 Aarhus, Denmark; Copenhagen Research Center for Biological and Precision Psychiatry, Mental Health Centre Copenhagen, Copenhagen University Hospital, 2900 Copenhagen, Denmark

**Keywords:** schizophrenia, developmental coordination disorder, specific language impairment, psychosis, bipolar disorder

## Abstract

**Background:**

It is known that impairments in linguistic ability and motor function tend to co-occur in children, and that children from families with parental mental illness such as schizophrenia tend to perform poorly in both domains, but the exact nature of these links has not yet been fully elucidated.

**Design:**

In this study, we leveraged the first wave of the Danish High Risk and Resilience Study (VIA 7), which includes both genetic data and measures covering multiple developmental domains. The VIA 7 cohort comprises 522 7-year-old children born to parents with schizophrenia (*N* = 202), bipolar disorder (*N* = 120) or neither (*N* = 200). We investigated the relationships between linguistic ability and motor function using correlation and regression analyses, focusing on developmental coordination disorder (DCD) and specific language impairment (SLI) and their potential associations with the three risk groups.

**Results:**

We found significant correlations between most measures of language and motor function and significant associations of DCD and SLI with language and movement measures, respectively, the largest effect being that of DCD on receptive language, with a significant interaction effect: DCD was associated with poorer performance in children from schizophrenia families compared to bipolar disorder and control families. Both disorders showed higher prevalence among children with familial high risk of mental illness. We did not find significant evidence of genetic overlap between DCD and SLI.

**Conclusions:**

Our results suggest strong links between the domains of motor function and linguistic ability. Children of parents with schizophrenia are at high risk of comorbid language and movement disorders.

## Introduction

Linguistic ability and motor function are essential facets of the child’s neurodevelopment, and disorders affecting one or both domains may contribute to scholastic problems, pose an obstacle to interacting with peers and developing friendships, and ultimately give rise to challenges with self-esteem and emotional health.^1–6^ Specific language impairment (SLI) is a neurodevelopmental disorder in which language development is significantly below the level expected for the child’s age and intelligence, in the absence of any obvious etiology, such as hearing impairment, intellectual disability, or neurological disorders.^7^ In recent years, however, the previous requirement for a discrepancy between verbal and nonverbal intelligence has been reevaluated, resulting in a new label, developmental language disorder (DLD), which is more inclusive.^8^ Similarly, developmental coordination disorder (DCD) is a neurodevelopmental disorder in which the child’s motor coordination does not meet expectations for their chronological age and IQ, and where difficulties in the coordination of either gross or fine motor movements (or both) interfere with academic achievement and/or activities of daily living.^9^ As with SLI, the motor difficulties that characterize DCD cannot be ascribed to an underlying medical condition or disease such as, for example, cerebral palsy, muscular dystrophy, visual impairment, or intellectual disability.^9^ Both SLI (together with DLD) and DCD have been shown to be heritable.^10–13^

While DCD and SLI are typically diagnosed during childhood, they may lead to persistent psychosocial difficulties and poorer mental health in adulthood, for example in the form of depressive and anxious symptoms in DCD.^14,15^ Individuals with DLD have similarly been found to have, on average, higher scores on the Schizotypal Personality Questionnaire in their mid-thirties compared to unaffected individuals, and the prevalence of a confirmed diagnosis of schizophrenia was larger among those who had DLD than in the general population; similarly, they reported high rates of difficulties in social adaptation.^16^ Adults with a schizophrenia-spectrum disorder have also been found to have exhibited significantly poorer premorbid motor coordination during childhood in comparison with unaffected controls and poorer premorbid coordination than those who were later diagnosed with a nonpsychotic mental illness in adulthood.^17^ The above studies point to high comorbidity, not just between neurodevelopmental disorders, but also between these and psychiatric disorders.

In this study, we used data from the Danish High Risk and Resilience Study (the VIA 7 study), a cohort comprising 522 7-year-old children with familial high risk of schizophrenia-spectrum disorder (FHR-SZ) or bipolar disorder (FHR-BP), or population-based controls (PBC).^18^ The children have been assessed on multiple developmental domains, including motor function and linguistic ability. Importantly, two previously published papers from the VIA 7 study have shown that both language deficits and motor deficits are associated with familial high risk of schizophrenia,^19,20^ and a follow-up study of the children reported higher odds of psychotic experiences in children scoring within the 5th percentile on a standardized movement test battery.^21^ In the case of schizophrenia, other studies have also reported finding potential disease precursors in several neurodevelopmental areas such as: cognition, motor function and behavior during childhood and adolescence.^22–24^ In these previous studies, however, linguistic ability, motor function and familial high risk of mental illness were not examined or modeled simultaneously, and potential interactions between them were not investigated. Furthermore, the genetic overlaps between disorders in those domains have not been investigated. With this in mind, our study attempted to answer three main questions, focusing both on the overlaps between movement and language disorders and traits, as well as on their links to familial high risk of schizophrenia and bipolar disorder:

1. Is there a phenotypic overlap between measures of motor function and linguistic ability?
2. Is there comorbidity between DCD and SLI, and could it be driven by a genetic overlap?
3. In what way is familial risk of schizophrenia or bipolar disorder associated with measures of motor function and linguistic ability as well as with DCD and SLI?

## Methods

### Participants

The Danish High Risk and Resilience Study (the VIA 7 study)^18^ is a prospective cohort consisting of 522 children with at least one biological parent affected by a schizophrenia-spectrum disorder (*N* = 202) or bipolar disorder (*N* = 120), and children of parents with neither disorder (PBC) (*N* = 200). The cohort was established when the children were 7 years old, and recruitment and data collection took place between January 31st, 2013 and January 21st, 2016. Eligible children were identified using Danish National Registries. Children at familial high risk of schizophrenia (FHR-SZ) were matched to PBC on age, sex, urbanicity, and geographical location as a proxy for socioeconomic status. Children at familial high risk of bipolar disorder (FHR-BP) were not matched but were similar to PBC with regard to age and sex. As reported in our previous study, some of the children presented symptoms compatible with a psychiatric diagnosis at the time of assessment.^25^ Three children were taking psychotropic medication (methylphenidate or atomoxetine, for the treatment of ADHD).

### Test Batteries and Procedures

We assessed the children’s motor function using the Movement Assessment Battery for Children, Second Edition (Movement ABC-2).^26^ The child’s fine motor skills (manual dexterity, *N* = 514) are assessed via three tasks: peg placing, threading lace, and drawing a trail. Praxis (aiming and catching, *N* = 514) is evaluated on catching a ball with both hands and throwing a beanbag onto a mat. Lastly, the domain of balance (*N* = 512) measures dynamic and static balance via three tasks: one-board balance, walking forward on a line (heel-to-toe), and hopping on mats. The test battery was carried out by a total of 11 raters (medical doctors, psychologists, and a nurse) who had all previously been trained by a physiotherapist authorized to administer the Movement ABC-2. In addition, homogeneity in the performance of the test battery and inter-rater variability were regularly assessed using videos of study participants doing Movement ABC-2. With rare exceptions, Movement ABC-2 was performed in the same two rooms at the research sites in Copenhagen and Aarhus. Raters were blinded to the child’s familial risk status at the time of the assessment. The scores from the subtests as well as the total score, were standardized as described previously.^20^ Language ability was measured using the Test for Reception of Grammar-2 (TROG-2, *N* = 518),^27^ which is administered by the rater and measures the child’s receptive language. In this test, children are presented with 20 blocks of four sets of pictures; in each set, only one picture corresponds exactly to the sentence that the rater says to the child. The child is then asked to choose the picture that captures the situation described by the sentence. If the child chooses the correct picture in each set, then the child will have passed the block. There are 20 blocks in the test, and the number of “passed blocks” is standardized using Danish norms to produce the final score. The children also underwent an intelligence screening (Reynolds Intellectual Screening Test—RIST, *N* = 518),^28^ which is made up of a verbal intelligence component (“Guess What?”) and a nonverbal intelligence component (“Odd-item Out”), which are converted to age-adjusted T-scores (mean = 50, SD = 10) that can be combined and converted to an index score (mean = 100, SD = 15). Scores on the RIST were standardized using Danish norms. This test was used in a prior VIA 7 study.^29^

In the cases of all standardized scores used in this study, a higher score indicates better performance. Note that the TROG-2 tests receptive language ability, assessing only certain grammatical structures, and thus, it can be viewed as measuring a component of receptive language. The “Guess What?” part of the RIST relies on both receptive and expressive language.

### Definitions of Language and Movement Neurodevelopmental Disorder Phenotypes

In line with the Movement ABC-2 manual and previous reports,^30,31^ children with significant motor difficulties, as indicated by having a Movement ABC-2 standardized total score at or below the 5th percentile, were defined as DCD cases. Cases were also required to have a RIST index score ≥ 70. DCD controls were defined as having a total standardized score above the 15th percentile. Children with a Movement ABC-2 score above the 5th percentile but at or below the 15th percentile were excluded from analyses with the DCD phenotype to ensure a clear distinction between DCD cases and controls; even though these children present some motor difficulties, we did not have sufficient clinical data to classify them as cases, and we did not want to misclassify them as controls. This has been the practice in in other studies,^32^ and the Leeds Consensus Statement of 2006 also recommends not to classify children in this range as DCD cases.^33^ SLI cases were defined as having a standardized TROG-2 score ≤ 77.5 (a score of 77.5 being 1.5 SD below the population mean of 100) while being required to have a RIST nonverbal intelligence (“Odd-item Out”) score ≥ 35. Moreover, SLI cases must not have had any indication of an autism spectrum disorder (ASD) from a semi structured child and adolescent psychiatric interview, The Kiddie Schedule for Affective Disorders and Schizophrenia for School-Age Children—Present and Lifetime Version (K-SADS-PL)^34^; children who were suspected of having ASD based on the assessor’s evaluation of the responses (provided by the child and primary caregiver) on the ASD sub-section of the screening interview of the K-SADS were administered the ASD supplement of the K-SADS. Subsequently, their results were discussed at a meeting between the assessor and a board-certified child and adolescent psychiatrist, where the indication of ASD would either be confirmed or rejected. This was done upon evaluation of the child’s and the caregiver’s responses to specific items of the K-SADS, anamnestic information provided by the caregiver, an assessment of the child’s global functioning, and direct clinical observation of the child by the assessor. SLI controls were defined as children who had a standardized TROG-2 score ≥ 92.5 (a score of 92.5 being 0.5 SD below the population mean of 100). These definitions correspond to the definitions used by the SLI Consortium in the discovery genome-wide association study (GWAS) for SLI,^35^ where SLI cases were defined as being a proband from an SLI family or having either an expressive language score or a receptive language score from the revised Clinical Evaluation of Language Fundamentals lower than 1.5 SD below the population mean while not having a low nonverbal intelligence score. Note that controls were not used in the GWAS (which used a specific family-based model and not case-control), but they were defined as having both expressive and receptive language scores above 0.5 SD below the population mean (i.e., above the value which is equal to the population mean minus half a standard deviation).^1^ We note that, since we did not have clinical language data or expressive language scores for the children, it is possible that some children classified as controls would have been classified as cases for the SLI phenotype, if there had been indications of SLI based on clinical language data or expressive language scores. In our sample, 117 children met our DCD case criteria, 329 children met our DCD control criteria, 33 children met our SLI case criteria, and 380 children met our SLI control criteria.

### Statistical Analyses

Statistical analyses were performed in R v4.2.2.^36^ The distributions of the test scores for each trait are shown in Supplementary Figure S1, which contains histograms and density plots for the traits and was generated in R using the *hist* and *density* functions. A plot for the mean score in the different groups was created with the *dotplot* function of the lattice package v0.21-8.^37^ We calculated the pairwise Pearson’s correlation coefficients across the traits in the sample of children with genotypes used in this study. This was done using the Hmisc package v5.0-1,^38^ and the plot was generated with the corrplot package v0.92.^39^ Linear regressions of the quantitative phenotypes on disorder affection status (language scores were regressed on DCD, and movement scores were regressed on SLI) with covariates for sex and familial high-risk status of mental illness (defined as an unordered factor with three levels: control family (reference level), schizophrenia family and bipolar disorder family) were performed with the *lm* function, and confidence intervals were calculated with the *confint* function. Boxplots were generated using the *boxplot* function with the default parameters. Fisher’s exact test was used to test for association between SLI and DCD, with the *fisher*.*test* function. For this test, we had 266 children with neither DCD nor SLI, 61 with only DCD, 10 with only SLI, and 19 children with both DCD and SLI. The odds ratio and 95% confidence interval for it were obtained with this function.

### Genetic Dataset and Polygenic Score Analyses

DNA samples were genotyped on the Illumina PsychChip v1-1_15073391_C. The full quality control steps for the genetic dataset were described in detail elsewhere.^40,41^ Our genetic analyses in this study are based on a polygenic score (PGS), sometimes called polygenic risk scores (PRS), for SLI. The SLI PGS was trained on a genome-wide association study of SLI,^35^ as described in our previous study.^42^ The PGS analyses were performed in a subset of the sample which included 391 unrelated VIA children who passed the genetic quality control, in line with our previous analyses.^40,42^ The SLI analysis included 21 cases and 288 controls; the DCD analysis included 83 cases and 256 controls; and the height analysis included 274 children with data for height and the covariates. Note, however, that the PGS used in this study was regenerated with a newer version of PRSice,^43^ namely v2.3.5; while the genetic dataset and the parameters for PRSice were the same, the newer version of the program had a modified algorithm which could lead to minor differences in the PGS calculation compared to previous versions. Therefore, we repeated the analyses for the SLI and height phenotypes. The PGS analyses for the SLI and height phenotypes in VIA were included as a positive control and a negative control, respectively, to assess the performance of the SLI-trained PGS; this means that we would expect the SLI-trained PGS to be associated with SLI affection status in VIA but not with height (a heritable trait not expected to be genetically correlated with SLI). The *P*-value threshold was pT = 1, the clumping parameters were an r^2^ of 0.2 and a window of 500 kbp, and the MHC region was removed from the target dataset. The regressions were performed by PRSice and the adjusted R^2^ was based on a prevalence of 7% for SLI^44^ and 6% for DCD.^45^ The analyses for height included two models: one with the PGS and covariates for sex and the age at which height was measured and one with only the covariates, and the R^2^ for the PGS in the height analysis was calculated as the R^2^ for the model with the PGS and covariates minus the R^2^ for a model with only the covariates. From the PRSice output, we used the PGS R^2^ value and (where applicable) the adjusted R^2^ value. The PGS was then standardized over the entire sample of children, such that the units of the PGS are SD from the mean PGS of the sample. Logistic regressions (SLI and DCD) or linear regressions (height) were performed in R v3.6.1 with the *glm* (family = binomial(link = “logit”)) and *lm* functions, respectively, to obtain the coefficients for the PGS reported here.

### Tests for Interaction Between Familial High Risk Status and Neurodevelopmental Phenotypes

We also repeated the linear regressions having added a term for an interaction between the disorder and the familial high-risk status. Thus, when, for example, regressing the TROG-2 score on DCD status + sex + familial high-risk status (as before), we added a term for an interaction between DCD and familial high-risk status. We tested whether adding this term improved the model using a likelihood ratio test using the *anova* function (test = “LRT”) with the two nested models, comparing the new model to the original one, without the interaction term. If the interaction term significantly improved the model, we performed another regression with the interaction term but without the term for the disorder itself (i.e., the disorder without the interaction). This results in reported coefficients for the disorder in the context of each familial high-risk group (control family, schizophrenia family, and bipolar disorder family), and we report the effect for the disorder in the relevant groups from this model.

### Likelihood Ratio Test for Model Improvement for SLI PGS

For the SLI phenotype in the target sample, we also performed a likelihood ratio test to see if adding the SLI-trained PGS to a model of the SLI outcome (in the VIA 7 cohort) regressed on the familial high-risk status (for mental illness) significantly improved the model, and vice versa. This was done using nested models in a likelihood ratio test (SLI regressed on familial high-risk status (or SLI-trained PGS); SLI regressed on familial high risk status and SLI-trained PGS) with the *anova* function (test = “LRT”).

## Results

In our study, 58% of the SLI cases also met criteria for DCD, as compared with only 16% of DCD cases meeting criteria for SLI. Furthermore, the SLI prevalence in the full sample was much closer to the prevalence of SLI in the general population, whereas the prevalence of DCD was about four times as high as the prevalence of DCD in the general population, even though the sample was not ascertained for either disorder. The prevalences of DCD and SLI among boys and girls in the study were ~2.3 times higher in boys than in girls. The highest proportion of cases with both SLI and DCD were found in the group of children with familial high risk of schizophrenia; ~11% of the children at familial high risk of schizophrenia presented both DCD and SLI, compared to ~4% of the children in the group at familial high risk of bipolar disorder and ~1% of controls (among children meeting DCD and SLI case or control criteria). Descriptive statistics for all phenotypes across the groups are found in Supplementary Table S1.

### Correlations Between Language Traits and Movement Traits in the Full Cohort

**Figure 1.**
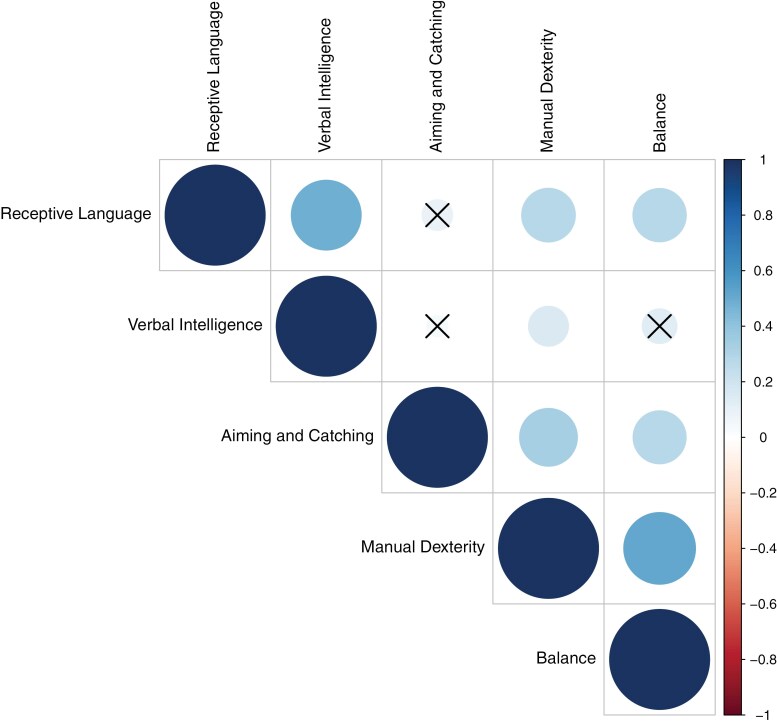
Pairwise Pearson Correlations Between Language (TROG-2 and RIST Verbal Intelligence) and MABC-2 Standardized Scores (i.e., the MABC-2 Total Standardized Score and the Standardized Score for Each MABC-2 Subdomain). The Correlations that are Crossed out did not Survive Bonferroni Correction for the Number of Tests

We found significant correlations between the children’s performance on most linguistic domains (receptive language and verbal intelligence) and motor domains (manual dexterity, aiming and catching, and balance), as shown in Figure 1. After Bonferroni correction for the number of pairwise comparisons, only the correlations between the aiming and catching subtest from the Movement ABC-2 and TROG-2 and RIST verbal intelligence and the correlation between the balance subtest of the Movement ABC-2 and RIST verbal intelligence were not significant. All other correlations were positive and significant at α = .05 after Bonferroni correction.

### Distributions of Scores Across Groups Stratified on Familial High Risk of Severe Mental Illness

**Figure 2.**
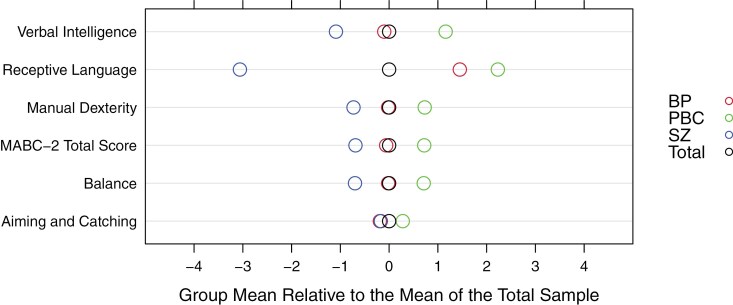
Difference in Group Means of the Quantitative Phenotypes Relative to the Mean of the Total Sample, Across Familial High-Risk Groups, and Controls. SZ: Schizophrenia Familial High-Risk Group; BP: Bipolar Disorder Familial High-Risk Group; PBC: Population-Based Controls

We observe lower scores, on average, in the FHR-SZ group compared to the total sample and controls across all movement and language measures, while the FHR-BP group performs closer to the total sample on most measures, but nonetheless poorer than controls. Figure 2 shows the differences between group means for all quantitative phenotypes.

### Cross-Domain Associations Between the Quantitative Phenotypes and the Disorders

**Figure 3.**
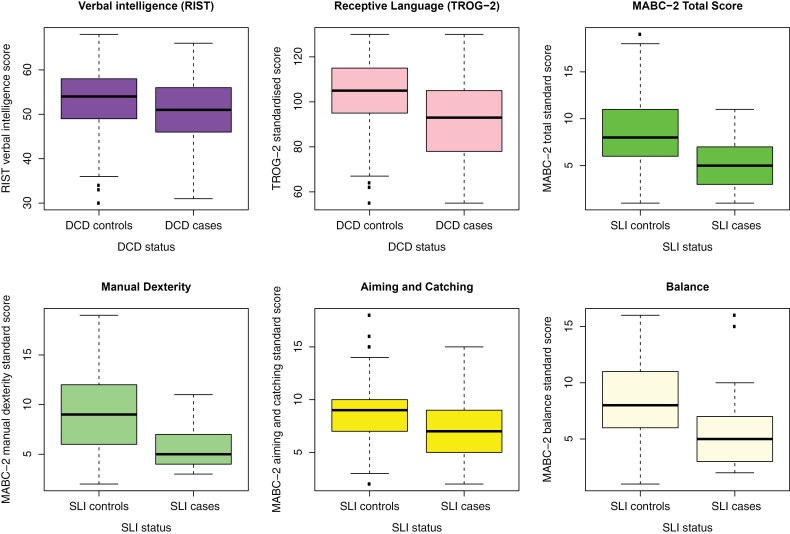
Box Plots for Language Scores (TROG-2 and RIST Verbal Intelligence) Among DCD Cases and Controls, and Standardized Scores Across the Three Subsets and the Total Standardized Score of MABC-2 Among SLI Cases and Controls, Generated With the *Boxplot* Function in R With Default Parameters. The Thick Line in the Middle Indicated the Median. The Whiskers in the Box Plots Indicate Data Extremes Without Outliers, While Dots Indicate Outliers. MABC-2: Movement Assessment Battery for Children, Second Edition; SLI: Specific Language Impairment; RIST: Reynolds Intellectual Screening Test; TROG-2: Test for Reception of Grammar, Second Edition. Please Note That the Figure is Meant to Show Descriptive Statistics and Trait Distributions; See Table 1 for Tests of Significance for the Effects of SLI and DCD on Movement and Language Scores, Respectively, Where Linear Regressions Were Performed With Covariates for Sex and Familial High-Risk Status

SLI cases had lower scores on the movement subtests than SLI controls, and DCD cases had lower language scores than DCD controls (Figure 3). We further examined these relationships using linear regressions while including covariates for the sex and the familial high-risk status of the child. Apart from the analysis for aiming and catching, the disorder always had a significant negative effect on the trait from the other domain (i.e., SLI on motor function and DCD on language ability) after Bonferroni correction (Table 1). The covariates for sex and familial high risk of schizophrenia were at least nominally significant in some of the regressions (for sex, in all but the regression for RIST verbal; for schizophrenia, in all but the regression for aiming, and catching), but familial high risk of bipolar disorder was not. In the post hoc tests, which included a term for an interaction between the disorder (SLI/DCD) and the familial high-risk status, only the regression with the TROG-2 score as the outcome was significant in the likelihood ratio test for the interaction (*P* = .0284). The effect of DCD on the TROG-2 score was significant in schizophrenia families (β = −14.90, standard error = 2.40, *P* = 1.32 × 10^−9^) and in bipolar disorder families (β = −11.19, standard error = 3.23, *P* = .0006). It was not significant in control families, but it was in the same direction (β = −5.02, standard error = 2.88, *P* = .0816). We also found a significant association between SLI and DCD themselves using Fisher’s exact test, with an odds ratio (OR) of 8.22 (two-sided *P* = 2.28 × 10^−7^; 95% confidence interval = [3.44, 20.85]).

**Table 1.** Results of the Linear Regression Analyses of Language and Movement Scores on DCD/SLI Status

Outcome	Predictor^a^	Estimate (β)	95% confidence interval	*P*-value (for the estimate)
RIST verbal	DCD	−2.53	[−4.05, −1.02]	.001
TROG-2	DCD	−10.94	[−14.15, −7.72]	6.96 × 10^−11^
MABC-2 total score	SLI	−2.74	[−3.88, −1.59]	3.83 × 10^−6^
MABC-2 manual dexterity	SLI	−2.47	[−3.67, −1.26]	6.72 × 10^−5^
MABC-2 balance	SLI	−2.34	[−3.55, −1.13]	1.69 × 10^−4^
MABC-2 aiming and catching	SLI	−1.10	[−2.16, −0.04]	.043

Abbreviations: DCD: developmental coordination disorder; MABC-2: movement assessment battery for children, second edition; SLI: specific language impairment; TROG-2: test for reception of grammar, second edition.

^a^All regressions included covariates for familial high risk of mental illness and sex.

### Genetic Overlap Between SLI and DCD

We checked the validity of the SLI-trained PGS by confirming that it was predictive of SLI itself in the VIA cohort, but not of height. The SLI-trained PGS was not predictive of DCD in the VIA cohort. In other words, we found no significant evidence for additive genetic effects which influence SLI risk also influencing DCD risk. Table 2 shows the full results of the analyses. Given that most children with SLI in the VIA study come from families with parental schizophrenia-spectrum disorders (FHR-SZ), we performed a post hoc likelihood ratio test for the improvement in SLI prediction in VIA using either only the familial high risk status or both the high risk status and the SLI-trained PGS, and found that despite the higher prevalence of SLI children among FHR-SZ families in our sample, adding the PGS significantly improved the model (LRT *P* = .048), with the Nagelkerke’s R^2^ increasing from 10.84% without the PGS to 13.93% with the PGS. Similarly, adding the familial high risk status to a model with only the PGS increased Nagelkerke’s R^2^ from 3.80% to 13.93% (LRT *P* = .002). Models with both variables separately or combined were also significantly improved compared to a model with only the intercept. In all cases, the coefficients for the PGS and familial risk of mental illness were positive. These results suggest that both genetic predisposition to SLI and familial high risk of mental illness increase SLI risk in the child.

**Table 2.** Results of the Polygenic Score Analyses

Phenotype	Estimate (β) for standardized SLI-trained PGS (corresponding odds ratio^a^)	Standard error of the estimate	R^2^^b^	Adjusted R^2^^c^	*P*-value (for the estimate)
SLI (positive control)	0.49 (1.63)	0.23	3.80%	5.52%	.033
Height (negative control)	0.14	0.54	0.02%	–	.792
DCD	0.17 (1.19)	0.13	0.82%	0.67%	.173

Abbreviations: DCD: developmental coordination disorder; PGS: polygenic score; SLI: specific language impairment.

^a^Only applicable to binary traits; odds ratios for logistic regressions were calculated as e^β^.

^b^Nagelkerk’s pseudo-R^2^ for binary phenotypes; R^2^ for height.

^c^Only applicable to binary traits; transformation with PRSice taking into account the prevalence and the proportion of cases in our study.

## Discussion

Our study identified significant, positive correlations between multiple motor subdomains and language ability. DCD and SLI were significantly positively associated with each other. DCD was significantly negatively associated with linguistic ability, and SLI was significantly negatively associated with motor function. Such phenotypic overlaps are in line with prior findings from the literature with regards to both the quantitative trait correlations and the concomitance of the disorders.^46^ Importantly, a meta-analysis of 16 smaller studies confirmed the general association between language impairment and motor impairment described in our study.^47^ The weaker association between SLI and a lower performance in aiming and catching is also consistent with prior findings in the literature.^48^

### Lack of Evidence for Genetic Overlap Between SLI and DCD

The lack of significant genetic overlap between the disorders could partly be due to small sample sizes in this study and/or in the discovery GWAS. A previous report suggested that genetic factors may be involved in the phenotypic overlap between motor and language impairment in the context of the procedural deficit hypothesis.^49,50^ An alternative scenario could be that the overlap between SLI and DCD is due to developmental neural pathways that lead to similar phenotypic outcomes, with each one being mostly associated with different genetic etiologies, a phenomenon called phenomimicry, where the causal route for one disorder can lead to an outcome resembling the other.^51^ Phenomimicry does not explain why not all children with SLI have DCD and vice versa, but assuming that the severity of one type of impairment influences the manifestation of the other may offer some explanation,^51^ and the high correlations between the scores on language and motor tasks are in line with this assumption. In Bishop’s survey of hypotheses regarding the supposed phenotypic overlap between SLI and ASD,^51^ she notes two problems that challenge the notion that phenomimicry could account for the overlap between SLI and ASD: (1) the observation that not all children with ASD have SLI-like language problems, and (2) the fact that some genes have been found to be involved in both conditions. But, in the case of SLI and DCD, these issues do not arise (with the above assumption regarding the severity of the impairment). A number of studies have identified candidate genes for SLI,^35,52–57^ but these are few compared to those of other neurodevelopmental disorders, such as ASD.^58^ There are even fewer genetic studies of DCD; a recent GWAS for DCD did not find genome-wide significant associations.^59^ Thus, there is currently no evidence for the implication of specific genes in both disorders. Interestingly, one gene, *SETBP1*, has been implicated in both DLD^60^ and schizophrenia,^61^ but PGS-based analyses have not found a significant overlap between the disorders.^42^

### Interplay Between DCD, SLI, and Familial Risk of Mental Illness

A bidirectional relationship between motor and language development has been reported in the literature.^46,62^ As concerns the difference in the prevalences of SLI and DCD between boys and girls, our findings are in line with the literature, where previous population studies on 7-year-old children reported a higher prevalence of DCD in boys than girls.^63^ For SLI, studies differ on the matter.^44,64–66^ Both disorders were more prevalent in children at familial high risk for schizophrenia in our study. While this is in line with previous findings for the quantitative language and movement measures,^19,20^ the concomitant presentation of DCD and SLI in this population is reported here for the first time. With the exception of the effect of DCD on TROG-2, SLI and DCD affected motor and linguistic traits (respectively) independently of familial risk of mental illness.

Correlations between language development and motor development have also been reported in the literature.^62,67^ Some authors hypothesize that the acquisition of motor skills in itself allows the child to gain skills that would, later on, be useful for language and communication.^62^ Others have argued that motor actions themselves are influenced by social cognition, especially in the interaction between mother and child during the performance of specific tasks.^68^ With regards to the association between deficits in the two domains, it has been shown that poorer motor performance makes the child a less attractive playmate.^69^ Hence, motor impairment may result in a more limited social interaction with peers, which could indirectly contribute to language impairment. Conversely, poor language and communication skills at an early age could also lead to the child’s having difficulty with peer interaction through play and physical activities,^70^ thus impairing normal motor development.^71^ The observed phenotypic overlap could, at least in part, be due to environmental influences on brain development brought forth by having either language impairment or motor impairment. It has also been shown that brain development is generally influenced by caregiver-infant interaction.^72^ This finding is particularly relevant, as we observed a higher prevalence of both DCD and SLI among children of parents with schizophrenia. In fact, mental health problems, medication, and hospital admissions have been reported to affect mothers’ ability to care for their child and may thus hamper mother-child interaction.^73^ Importantly, specific adverse experiences, such as neglect and trauma in childhood, have been shown to be associated with smaller cerebellar volumes^74,75^; the cerebellum is known to be involved in motor coordination across many species,^76^ but emerging evidence suggests that, in humans, it is also involved in language.^77–80^ Recent studies have found an association between a gene highly expressed in the cerebellum, *NFXL1*,^81^ and SLI or other disorders involving language.^55,82,83^ Studies have also reported that major white matter tracts involved in language, such as the superior longitudinal fasciculus, may present anomalies in myelination, axon integrity, or axonal architecture in individuals with schizophrenia, and that these differences may be most significant at a younger age and tend to decrease as the brain reaches full development.^84^ Other shared neural pathways between linguistic ability and motor function have also been described.^85^ On a clinical level, reduced spontaneous hand gestures and a mismatch between expressive language and gestures have been observed in both affected patients and at-risk individuals,^86^ indicating a possible convergence of language and motor deficits that may overall affect communicative skills.

### Importance of the Findings With Relation to Diagnosis and Intervention

Our study shows that environmental factors are likely driving at least some of the association between language ability and motor function. Furthermore, in the case of SLI, we showed that familial risk of mental illness and genetic predisposition to SLI both improved the model of SLI risk compared to having only one of them in the model, and that DCD had a larger effect on receptive language in schizophrenia families. These findings, given the lack of significant evidence for genetic overlap between SLI and schizophrenia,^42^ suggest that the effect of parental mental illness on language is due to the home environment. If this is true, then children who have movement deficits and whose parents have schizophrenia might be at a higher risk of having language deficits because of both their home environment and their peer environment. This could explain the interaction effect we observe in this group of children, if the two risk factors worsen each other. At the same time, this provides the possibility of intervention in order to improve the outcome in those children. Given our findings, we would like to emphasize the importance of identifying the prognostic triad described previously^87,88^ i.e., genetic risk of schizophrenia, DCD, and the risk of psychotic symptoms in adolescence for a timely diagnosis of severe mental illness in children and adolescents, while advocating for a greater focus on the presence of language deficits. We recommend that children of parents with schizophrenia who show impairment in one domain be referred to specialists in both domains, as improvement in one domain could potentially lead to improvement in the other domain. This is important in countries where some of the support services are offered by the healthcare system while others are offered within the schooling system, and the two systems are not well integrated, as is the case in Denmark.^89^

### Strengths and Limitations

The major strengths of this study are that it had one of the largest sample sizes among studies on the topic,^47^ and that we investigated both the phenotypic and genetic relationships between language ability and motor function. However, it should be emphasized that, with regards to the genetic analyses, our sample size was not very large, and, therefore, there could be some genetic overlap, which our study was not powered enough to detect. It should also be mentioned that the DCD and SLI phenotypes in this study were defined on the basis of standardized test scores and not a clinical evaluation. The method of ascertainment of the disorder may, for instance, affect heritability estimates.^90^

The strong phenotypic overlap between SLI and DCD observed in our study suggests that early interventions for one of the disorders may have a positive effect on the other disorder. This is particularly important in the context of children at familial high risk of schizophrenia, who could be more susceptible to having DCD and SLI, in addition to their already increased risk of developing a psychiatric disorder.

## Supplementary Material

Supplementary material is available at https://academic.oup.com/schizophreniabulletin/.

sbae181_suppl_Supplementary_Figures_S1

sbae181_suppl_Supplementary_Tables_S1

## Data Availability

Access to the dataset used in the current study may be granted upon reasonable request to the principal investigators of the VIA project (https://viaundersøgelsen.org).

## Footnotes

1 Note that, while controls were not used in the GWAS in that study, there was a minor error in the definition for SLI controls in the text (in addition to the criterion of their not being probands); namely, it stated that controls had to have both language scores above the population mean. However, in practice, they were defined as having both scores above the value which was 0.5 SD below the population mean. Since the GWAS software used in the GWAS treated controls and individuals with an unknown affection status in the same way, and neither group was included in the GWAS, this did not make any difference. See, for example, Fabiola Ceroni: Investigating the role of Copy Number Variants in Specific Language Impairment and identification of new candidate genes (dissertation), Alma Mater Studiorum Università di Bologna, 2014. For the purpose of this study, we adopted the cutoff values used by the SLI Consortium, as in our previous studies.
