# Supplementary material for: Language, Motor Ability and Related Deficits in Children at Familial Risk of Schizophrenia or Bipolar Disorder
Source: Schizophr Bull. 2024 Oct 28;51(6):1555–67. doi: 10.1093/schbul/sbae181 (PMC12597494; doi:10.1093/schbul/sbae181)
Supplement: sbae181_suppl_Supplementary_Figures_S1 [file sbae181_suppl_supplementary_figures_s1.pdf]

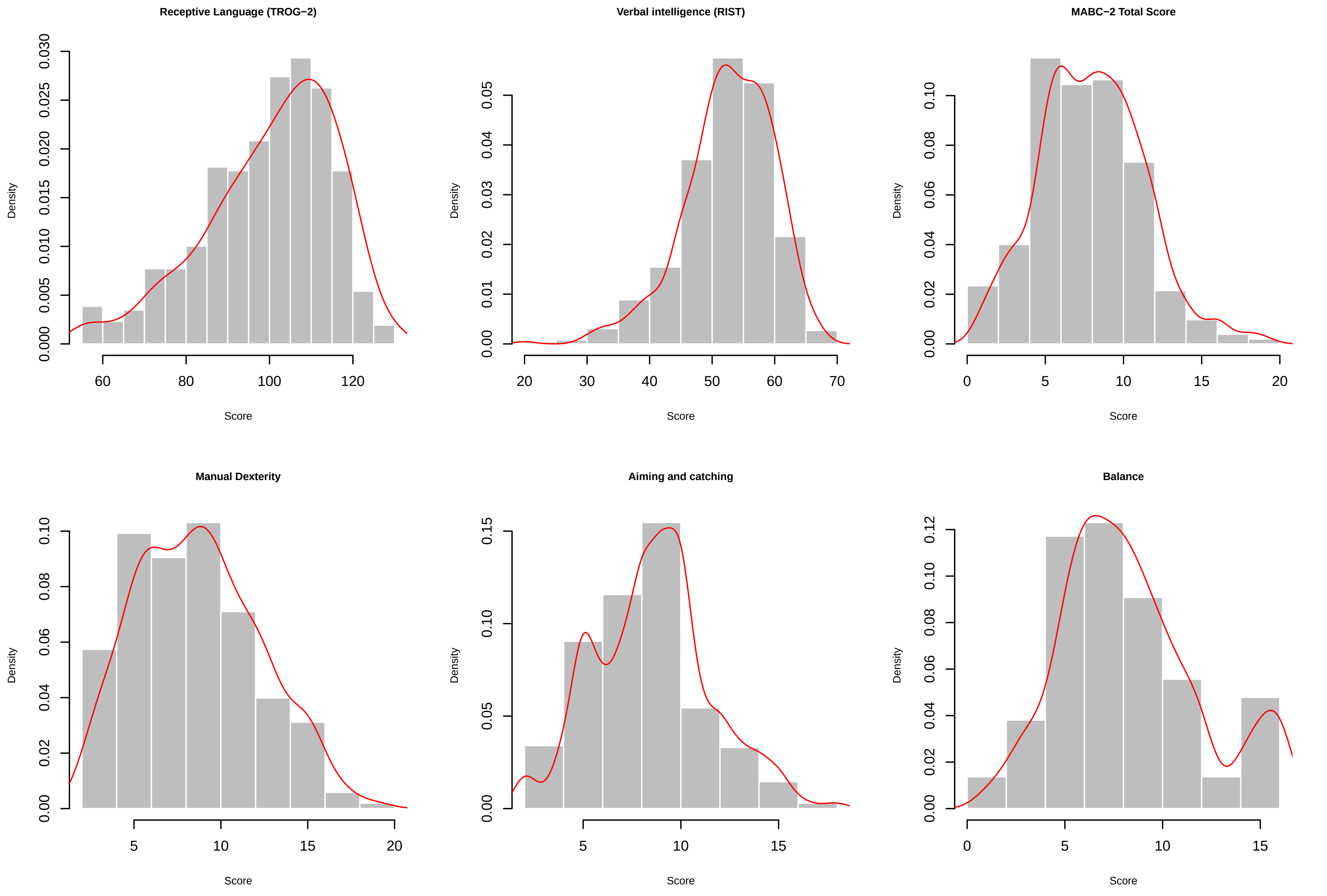

Receptive Language (TROG−2)
Verbal intelligence (RIST)
MABC−2 Total Score
Manual Dexterity
Aiming and catching
Balance
Density
Score
