# Supplementary material for: Language, Motor Ability and Related Deficits in Children at Familial Risk of Schizophrenia or Bipolar Disorder
Source: Schizophr Bull. 2024 Oct 28;51(6):1555–67. doi: 10.1093/schbul/sbae181 (PMC12597494; doi:10.1093/schbul/sbae181)
Supplement: sbae181_suppl_Supplementary_Tables_S1 [file sbae181_suppl_supplementary_tables_s1.pdf]

Supplementary Table S1: Descriptive statistics for the cohort

High risk group	TROG-2 receptive language score: mean (SD)	RIST verbal intelligence score: mean (SD)	MABC-2 total score: mean (SD)	MABC-2 manual dexterity score: mean (SD)	MABC-2 aiming and catching score: mean (SD)	MABC-2 balance score: mean (SD)	SLI case;control count	DCD case;control count
Total sample	100.63 (15.71)	52.67 (7.10)	8.01 (3.34)	8.71 (3.57)	8.59 (2.98)	8.33 (3.53)	33;380	117;329
SZ	97.57 (17.95)	51.58 (7.93)	7.32 (3.14)	7.98 (3.44)	8.42 (2.92)	7.63 (3.22)	24;132	56;115
BP	102.08 (13.78)	52.57 (6.51)	7.95 (3.26)	8.69 (3.56)	8.40 (3.06)	8.31 (3.44)	4;90	29;71
PBC	102.86 (13.81)	53.83 (6.38)	8.73 (3.45)	9.44 (3.57)	8.87 (2.99)	9.04 (3.75)	5;158	32;143

Individuals with missing phenotypes were excluded from the relevant calculations.
